# IoT Resource Allocation and Optimization Based on Heuristic Algorithm

**DOI:** 10.3390/s20020539

**Published:** 2020-01-18

**Authors:** Arun Kumar Sangaiah, Ali Asghar Rahmani Hosseinabadi, Morteza Babazadeh Shareh, Seyed Yaser Bozorgi Rad, Atekeh Zolfagharian, Naveen Chilamkurti

**Affiliations:** 1 School of Computing Science and Engineering, Vellore Institute of Technology (VIT), Vellore 632014, India; arunkumarsangaiah@gmail.com; 2 Deportment of Computer Engineering, Islamic Azad University Behshahr Branch, Behshahr 511-48515, Iran; A.R.Hosseinabadi@iaubeh.ac.ir (A.A.R.H.); At.Zolfagharian@gmail.com (A.Z.); 3 Department of Computer Engineering, Islamic Azad University, Babol Branch, Babol 47179-95449, Iran; Bozorgi@baboliau.ac.ir; 4 Department of Computer Science and Computer Engineering, La Trobe University, Melbourne VIC 3086 Australia; N.Chilamkurti@latrobe.edu.au

**Keywords:** internet of things, resource allocation problem, whale optimization algorithm, communication cost

## Abstract

The Internet of Things (IoT) is a distributed system that connects everything via internet. IoT infrastructure contains multiple resources and gateways. In such a system, the problem of optimizing IoT resource allocation and scheduling (IRAS) is vital, because resource allocation (RA) and scheduling deals with the mapping between recourses and gateways and is also responsible for optimally allocating resources to available gateways. In the IoT environment, a gateway may face hundreds of resources to connect. Therefore, manual resource allocation and scheduling is not possible. In this paper, the whale optimization algorithm (WOA) is used to solve the RA problem in IoT with the aim of optimal RA and reducing the total communication cost between resources and gateways. The proposed algorithm has been compared to the other existing algorithms. Results indicate the proper performance of the proposed algorithm. Based on various benchmarks, the proposed method, in terms of “total communication cost”, is better than other ones.

## 1. Introduction

Today’s world is at the beginning of the period of communication and universal processing. In this period, society is guided towards the always connected pattern. With the introduction of new technologies, the coverage of networks has been widened and the number of smart objects that are connected to the network has also been increased. A new pattern called the Internet of Things (IoT) increases the amount of information generated on the Internet through connecting people and smart objects. Thus, the IoT creates new challenges by connecting things and humans that we may face at any time and place [1].

The new generation of IoT has been created to achieve quality of service (QoS). Bandwidth is the most important source in the IoT environment, and its management is necessary for higher QoS. Moreover, the demand for multimedia services in the IoT environment has been increased dramatically. Different services provided in the IoT environment have different features and require different QoS methods [2,3]. The idea behind the IoT is to connect everything to each other through the internet [4,5]. It seems that there will be many applications in the future, and one of them is the smart city [5,6,7,8]. The use of the IoT will be seen everywhere, in food, clothing, housekeeping, transportation, education, entertainment, and so on. For example, monitoring the urban traffic situation and choosing the right strategy to control traffic lights represents one of the most well-known uses of the IoT [9]. Since IoT is used in many areas, many researchers have entered this field. Regardless of the type of research on the IoT, the main purpose is to improve human life. The first studies on the IoT are referred to Ashton’s paper [10]. Other papers have worked on sensor design, home health systems, smart grid systems, and so on, which are somehow associated with IoT [11,12,13].

To understand the importance of IoT, it should be mentioned that, according to the estimates, the number of connected devices in the IoT will reach 16 billion by 2021, and this industry will create a market of $900 billion by 2022 in America [14,15]. Although these values are just a prediction of the future of the IoT, what will undoubtedly occur is the expansion of the market and IoT in future decades.

The development and advancement of the IoT is not only focused on the development of hardware. Instead, a lot of work has been done to improve the efficiency of these systems. Many of these researches are trying to improve the efficiency of IoT-based systems by providing software methods. One of these problems is the IoT resource allocation problem (IRAP). This focuses on reducing the communication cost of IoT nodes. By reducing the communication cost in IoT, the efficiency of all related systems, such as RFID (or Radio Frequency Identification) and 5th generation wireless communications (5G), will increase. There are two types of nodes in each IoT system. The first type of node is the resource node. Each resource node is responsible for sensing the environment and sending data to other parts of the system. An IoT system has a lot of resource nodes that will enable it to monitor the whole environment. The second type of node in the system is the gateway node. A gateway node is responsible for directing traffic toward different resource nodes. In other words, a gateway node acts like a bridge for connecting resource nodes. An IoT system uses a limited number of gateways in order to connect resources. In such a system, the problem is which group of resources should be connected to each particular gateway in order to reduce the whole communication cost.

In an optimal solution, resources that pass many messages would be assigned to a single gateway. By means of this assignment, traffic between resources would not cause traffic between gateways. In other words, most of the traffic could be controlled by a single gateway. If we have a huge amount of traffic between gateways, there will be a bottleneck in gateway level communication [16,17,18,19,20].

In recent years, evolutionary algorithms have been widely used for solving optimization problems. These algorithms, by inspiration from nature or a physical phenomenon, attempt to optimize the solution of hard problems. Evolutionary algorithms are used for problems for which there are no solutions in polynomial time. Using good heuristic functions makes the evolutionary algorithm converge to the final solution at an acceptable time [21].

This paper focuses on solving the IRAP problem. In other words, the ultimate goal of this study is to achieve an optimal allocation pattern by providing a new method for solving the IRAP problem and a robust and new evolutionary algorithm called the whale optimization algorithm (WOA) has been used to solve the problem. The whale algorithm was proposed by Mirjalili in 2016, and is inspired by the collective whale hunting method [22,23,24].

The structure of the paper is organized as follows: Section 2 describes the related works. The WOA is explained in Section 3. The proposed algorithm is introduced in Section 4. Simulation results and conclusions are presented in Section 5 and Section 6 respectively.

## 2. Related Work

In recent years, many works have been done on binding physical resources to services in the IoT environment and other pervasive processing environments. In these environments, a physical resource called a smart object is selected to run a service. A number of articles focus on discovering a service based on resources and providing them to users. However, these articles do not work on resource allocation (RA) because resources and services are directly connected [25,26]. In this section, some papers devoted to RA are reviewed. The most important reviewed papers have been listed in Table 1. These papers can be divided into two categories. The first one is based on deterministic algorithms. These methods are old and not widely used todays. The second one is based on heuristic and evolutionary algorithms and are widely used today. In the following, we investigate them separately.

### 2.1. Methods Based on Deterministic Algorithms

Most RA papers in the IoT field focus on NP-hard problems. One of the old methods to solve this problem is deterministic algorithms. In these algorithms, a set of rules is designed for recourse allocation. These rules should balance between efficiency and effectiveness. Searching in the whole solution space requires a lot of time, on the other hand, less searches will cause poor resource allocation. The main advantage of the deterministic algorithms is the simplicity of their implementation [27,28].

In some papers, a comprehensive approach is provided which performs RA by resource management module in the middleware layer. In these methods, a centralized architecture is used which is not appropriate for the distributed structure of the IoT. It is also assumed that there is a static and reliable relationship between resources. Therefore, no attempt has been made to reduce the amount of data sent on the network [29,30]. In other research, authors have proposed a rule-based approach, in which some rules are designed for the size of the service, the completion time of tasks, and the capacity of the Virtual Machine (VM). The capacity of the VM is assigned to IoT services according to the designed rules [31]. Also, in one research two deterministic algorithms are proposed, each of them perform the RA according to a specific rule. In the first method, the average cost of each source is calculated and assigned to the most demanded service. In the second method, the most demanded resource is assigned randomly [32].

In some papers, game theory has been used for RA. For example, in [33], a game theory-based approach is proposed for tool-to-tool communication. This connection is created in a cloud-centered IoT environment. In this method, a response function is designed for RA. This function is designed such a way that increases the total profitability of the RA method. Kim et al. proposed another game-based approach to manage RA in the IoT. In this method, RA is done in such a way as to increase the total profit of the system [34].

The main problem of these methods is computational complexity. Given the fact that most of the RA problems are NP-complete and do not have a polynomial solution, solving these problems by means of a deterministic algorithm would be challengeable. In most of the above articles the main limitation of the proposed method is problem size. So many researchers in recent years have focused on heuristic algorithms, such as genetic algorithm, particle swarm optimization, and so on.

### 2.2. Methods Based on Heuristic Algorithm

Using heuristic algorithms is another way of solving the RA problem. This method has attracted many researchers in recent years. In heuristic algorithms, the main goal is to achieve an optimal solution without searching the whole solution space. Although the implementation of these methods is very complicated, they will give good results. In many cases, heuristic methods produce better results than deterministic ones. Heuristic algorithms are much faster than deterministic ones because they do not require a full search in the solution space. That’s why the use of heuristic algorithms has increased in recent years.

The genetic algorithm (GA) is one of the evolutionary algorithms used to design a heuristic method for RA. In this method, each chromosome represents a RA model. The fitness of each chromosome shows the quality of the scheduling. The evolution of the chromosomes with the help of genetic operators causes an optimal pattern for RA [35]. Kim et al. [36] used another form of GA, in which each chromosome is divided into two parts: gateway and resource. In this way, generated solutions can also reduce the cost of communication between gateways [36]. The particle swarm algorithm (PSO) is also used for RA. In this method, each particle represents a RA model. Particle motion based on the PSO algorithm will produce an optimal solution [37]. Other evolutionary algorithms such as simulated annealing (SA) [38,39], Tabo Search (TS) [40] and ant colony optimization (ACO) [41] are also used to solve the RA problem. In all of these methods, first, a number of random solutions are generated and then converted to an optimal solution in an evolutionary process. In some papers, the combination of several algorithms has been used to solve the RA problem. Combining several algorithms can lead to better solutions, because each algorithm covers the weakness of other ones. For example, in [42], the GA and ACO are combined to provide better solutions for RA problem. In [16], the search economics algorithm is combined with the k-means clustering algorithm to generate the Best Solutions (BS) in the shortest time. This method has focused on the IRAP problem. In recent years the RA problem has been tackled by deep learning-based approaches. Deep learning is suitable for problems with large scale data [43,44]. Singh et al. [45] proposed a new RA method to meet the service level agreements (SLA). They analyzed their method by means of two different metrics. One of them was capacity and the other one was enforcement period. They used buffering, scheduling and rate limiting to have a better RA. Authors in [46] see everything as resources and they illustrated their idea through RA in internet of things. Their case study was a healthcare-driven system that was based on IoT. They proposed a new RA method for such a system. The evaluation of the proposed method which is called IoTR4HealthCare system was benchmarked against two existing systems using cost and latency criteria [46]. Hatti et al. [47] proposed a RA algorithm based on fuzzy classification of jobs. In their method they improved a fuzzy interference system (FIS) for classification of the jobs into emergent and non-emergent. The job values should be sent to a global CPU to request resources from the cloud. Global CPU evaluates the resource need and free resources in terms of communication and computational cost. Based on the type of the job, a global CPU allocates resources by considering communication and computational cost. Dou et al. [48] proposed a new method which improved a RA algorithm with anti-jamming for the IoT nodes. In this method a novel automatic control allocation (ACA) model has been invented in order to provide an adaptive allocation and anti-jamming transmission. Moreover, the spreading-time technology is applied to fulfil the power and bandwidth requests of a node. In the algorithm, a new joint stepwise recursive function has been used to allocate resources in anti-jamming situation.

There are some problems in previous methods that make us motivated to propose a new method for this problem. The first problem is, there are many different versions of RA problem in the literatures, but just a few of them are suitable for IoT environment. So, this paper tries to present a new method that is adjusted for RA in IoT. The second problem is the inappropriate exploring of the problem space that can be seen in many papers. So, this paper tries to use a powerful heuristic algorithm in order to boost the exploration process. This algorithm that has been introduced in recent years is called WOA.

## 3. Formal Definition of IRAP

Before describing the proposed algorithm, the RA problem should be defined. In the problem mentioned in this study, there are two types of nodes. The first type is resources that are provided to service instances. The second node is gateways that are connected to resources. Gateways connect different parts of the IoT system. Each gateway is responsible for controlling the traffic of a number of resources. Resources can connect to different gateways. The cost of communication between a gateway and all resources is predetermined [16].

One aspect of the problem is that the resources are distributed between gateways, such a way that least cost of communication is caused.

**Figure 1:**
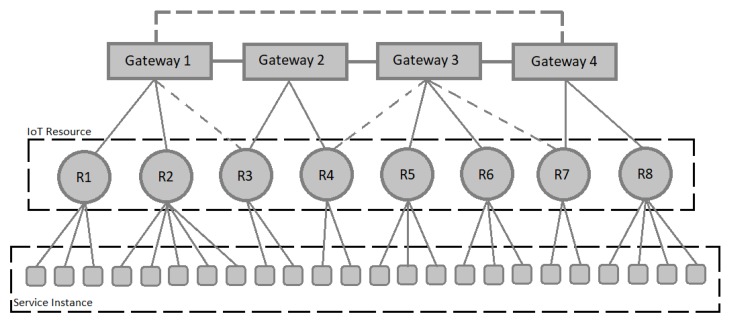
A solution for the IRAP problem

The cost between gateways is also an important part of the total cost. So, the other aspect of the problem is the kind of gateways’ connection to each other. Because of the high cost of communication between gateways, each solution of the problem connects gateways with the least number of connections. This connection will be in the form of a bus or ring. Figure 1 shows an example of the connection of resources and gateways. By changing the communication model, the cost of communication also varies. One of the goals of the problem is to find a pattern for RA that has the lowest cost of communication.

Another aspect is the load balancing in the IoT system. Load balancing means that resources are logically divided between gateways. Gathering all resources on a gateway will cause a bottleneck in the system. The way for calculating cost and load balancing is described in the objective function section. The problem discussed in this article is very similar to the scheduling problem mentioned in [16,36]. In the following section, objective function and problem variables are defined.

### 3.1. Objective Function

In a cloud computing environment, it is assumed that all resource nodes must communicate with each other. Therefore, for each solution of the RA problem, the total cost of network communications should be calculated. It is assumed that each resource sent a message to all other resources. The total cost of these messages are calculated and considered as objective function. The function named total cost and denoted as $T_{c}$. The proposed algorithm tries to minimize this function. Equation (1) calculates $T_{c}$.

(1) $$T_{c}=\frac{\sum_{j=1}^{\left|V_{g}\right|}\left(d_{j}^{r}\times d^{g}\right)}{p}$$

where $\left|V_{g}\right|$ is the total number of gateways, $d_{j}^{r}$ is the total cost of transferring data between *j*th gateway and all resources connected to it and $d^{g}$ is the total cost of communication between gateways which is calculated using Equation (2). Each inside gateway communication may cause inter gateway messaging. Hence, communication cost inside a gateway has an exponential effect on total communication cost. So, $\left(d_{j}^{r}\times d^{g}\right)$ is the best equation for calculating maximum value of the communication cost (objective function). It should be mentioned that the objective function value is supposed to be minimized [16]. In other words, we should multiply $d_{j}^{r}$ in $d^{g}$, because it is assumed that every single gateway may send a message to every single resource. So, by multiplying these two values, we can calculate the total communication cost for all resources of a gateway. If we calculate this value for all gateways, we would have numerator part of $T_{c}$ fraction.

(2) $$d^{g}=\overset{\left|V_{g}\right|}{\underset{i=1}{\sum }}\overset{\left|V_{g}\right|}{\underset{\begin{array}{c} j=1\\ j\neq i \end{array}}{\sum }}l_{ij}$$

where $l_{ij}$ is the cost of communication between gateways *i* and *j*. $d_{j}^{r}$ is calculated using Equation (3).

(3) $$d_{j}^{r}=\overset{\left|v_{g}^{j}\right|}{\underset{k=1}{\sum }}\varepsilon_{jk}$$

where $\varepsilon_{jk}$ is the cost of communication between *j*th gateway and all resources connected to it. $\left|v_{g}^{j}\right|$ is the number of connected resources to gateway *j*.

Another part of the fitness function is penalty P which is considered for balancing in RA. Given that, the proposed algorithm should reduce $T_{c}$, and P is the denominator of this function so $p_{i}=0$ indicates penalty and $p_{i}=1$ indicates normal mode in gateway *i*. The value of *p* is calculated for all gateways. For gateways having resources more than |Vr||Vg|, $p_{i}=0$ is mentioned to increase the value of $T_{c}$. Equation (4) calculates *p*.

(4) $$P=1+\overset{\left|V_{g}\right|}{\underset{i=1}{\sum }}P_{i}$$

where $\left|V_{g}\right|$ is the total gateways and $p_{i}$ is the penalty of each gateway which is calculated using Equation (5).

(5) $$P_{i}=\{\begin{array}{ccc} 1 & if & g_{i}^{t}\leq \varepsilon \frac{\left|V_{r}\right|}{\left|V_{g}\right|}\\ 0 & if & g_{i}^{t}>\varepsilon \frac{\left|V_{r}\right|}{\left|V_{g}\right|} \end{array}$$

In which, $g_{i}^{t}$ is the number of resources assigned to gateway *i*, $\left|V_{r}\right|$ is the number of resources and ε is a constant number. In order to simplify understanding this section, all notations and their meaning listed below:$T_{c}$: Total cost (the objective function)$\left|V_{g}\right|$: Total number of gateways$\left|V_{r}\right|$: Number of resources$\left|v_{g}^{j}\right|$: Number of connected resources to gateway *j*$d_{j}^{r}$: Total cost of transferring data between *j*th gateway and its resources$d^{g}$: Total cost of communication between gateways$g_{i}^{t}$: Number of resources assigned to gateway *i*$\varepsilon_{jk}$: Cost of communication between *j*th gateway and all resources connected to itP: Total value of penalty$p_{i}$: Penalty value for *ith* gatewayε: Constant number

## 4. Whale Optimization Algorithm (WOA)

The WOA is a novel meta-heuristic algorithm that is inspired by the Humpback whales [22]. In this algorithm, the optimization process starts with producing a randomly generated population for whales. These whales try to find the prey’s (optimum) location, then append (optimize) them by the encompassing or bubble-net method.

In the encompassing method [22], the Humpback whales improve their current location according to the best location as follows:

(6) $$D=\left|C⊙X^{*}\left(t\right)-X\left(t\right)\right|$$

(7) $$X\left(t+1\right)=X^{*}\left(t\right)-A⊙D$$

where *D* is the distance between the position vector of both prey $X{\left(t\right)}^{*}$ and whale X(t), ⊙ is an element-by-element multiplication and *t* is the current iteration number. *A* and *C* are coefficient vectors, and are defined as follows:

(8) A=2a⊙r−a

(9) C=2r

where *r* is a random vector as length of *X*, each index of *r* includes a random number in [0,1], and the value of a is decreased linearly from 2 to 0 in the iterations.

The bubble-net method can be performed in two ways. The first is the shrinking encompassing in which, the value of a in Equation (8) and *A* are decreased. The second is the spiral updating position, which is inspired by the helix-shaped movement of humpback whales around prey:

(10) $$X\left(t+1\right)=D^{\prime }⊙e^{bl}⊙\cos\left(2\pi l\right)+X^{*}\left(t\right)$$

where $D^{\prime }=\;\left|X^{*}\left(t\right)\;-\;X\left(t\right)\right|$ is defined as the distance between the whale and prey, *b* is a constant value used for specifying the logarithmic spiral shape, and *l* is a randomly generated value ∈[−1.1].

The whales can swim around the victim through a shrinking circle and along a spiral-shaped path concurrently:

(11) $$X\left(t+1\right)=\{\begin{array}{cc} X^{*}\left(t\right)-A⊙D & if\;p<0.5\\ D^{\prime }⊙e^{bl}⊙\cos\left(2\pi l\right)+X^{*}\left(t\right) & if\;p\geq 0.5 \end{array}$$

where *p* ∈ [0,1] is a random value for describing the probability of taking either the shrinking encompassing method or the spiral model to set the whales position. In the discovery phase, the Humpback whales explore for prey with a random manner. The position of a whale is set by specifying a random search agent rather than the best search agent as below:

(12) $$D=\left|C⊙X_{rand}-X\left(t\right)\right|$$

(13) $$X\left(t+1\right)=\left|X_{rand}-A⊙D\right|$$

where $X_{\mathrm{rand}}$ is a position specified randomly among the current population. The first algorithm depicts the total structure of the WOA.

## 5. The Proposed Algorithm

In terms of administration in IoT, less latency and energy consumption are two vital aspects of the network. Hence, in order to achieve these objectives, this paper has focused on the RA problem in IoT. As mentioned before, the main objectives of RA are reducing communication cost and load balancing. Reducing communication cost induces less latency in the network. By load balancing, we could eliminate any possible bottleneck from the network and increase the performance of the network. If we manage to meet these goals, there will be more opportunities for using this technology. In this paper, a new particle-based algorithm has been used to tackle the problem.

Heuristic algorithms have been widely used to solve complex optimization problems. Given that the RA problem is an NP-complete problem, heuristic algorithms can be used to find an optimal solution for that. In this paper, a new heuristic technique called WOA is used to solve the RA problem in cloud computing. The whale algorithm is inspired by the collective hunting method of a kind of Whale named the Humpback Whale. In the following we will investigate the details of WOA to solve RA problem.

### 5.1. Steps of the Proposed Algorithm

The proposed algorithm begins by creating a number of whales using ‘whalecreat’ function. Each whale represents a random solution for the scheduling problem. After this step, the fitness of each whale is calculated, and the BS is considered as the current optimal whale. Fitness function is equal to the total communication cost in the RA pattern of a whale. This function is named ‘fitness’ and explained in the following sections. After this step, whales begin to move. For each whale, the values of a , A , C , l , p are updated. A and C are constant coefficients. a∈[2.0] is a descending number. p∈[2.0] and l∈[2.0] are random numbers. The use of these numbers is explained in the following sections.

**Figure 2:**
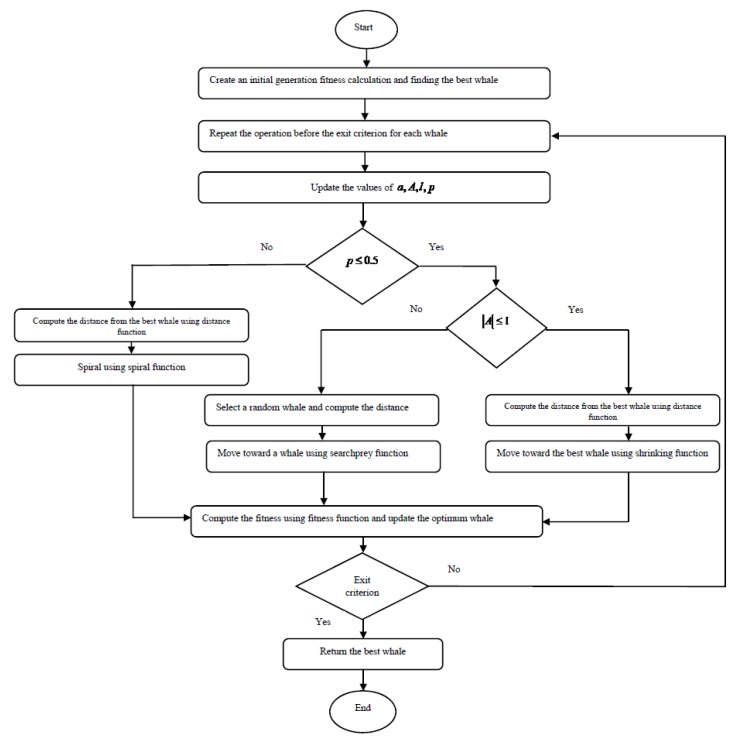
Flowchart of the proposed algorithm.

One of the most important functions in the whale algorithm is the distance between two whales. Since the whale algorithm is designed for a continuous problem and the RA problem is a discrete problem, this function needs to be rewritten. This function is used as ‘distance’ function in the algorithm. In total, three functions are designed for whale motion. The first one is “shrinking” function that reduces the distance between the current whale and the best whale. The second one is “spiral” function that simulates the current whale spin around the best whale. The third one is “searchprey” that moves a whale toward a random whale. The flowchart of the proposed algorithm is shown in Figure 2.

### 5.2. Whale Creating

In this algorithm, each whale represents a solution to the problem of RA. There are two types of nodes in the mentioned problem: gateway and resource. Each gateway is assigned a number of resources, and their information is sent just to this gateway. On the other hand, gateways are connected based on a specific topology in order to create the complete flow of information in the cloud. Due to the cost of data transmission between the gateways, their connection is created with the least edges and in the form of a spanning tree or a ring. Accordingly, each whale represents two sets of communications: edges that show the connection between gateways and edges that show the resources assigned to each gateway. A one-dimensional array w of size k+n is used to display a whale, where *k* is the number of gateways and *n* is the number of resources. The first *k* entries of array *w* (w[1.k]) show the relationship between gateways. w[i]=j means that gateway *i* sends its information to gateway *j* (i≠j). The second *n* entries of array *w* (w[k+1.n]) represent gateway of each resource. w[p]=q indicates that resource p−k sends its information to gateway *q*. Table 2 shows an example of how the whale is displayed in the proposed algorithm. There are four gateways and seven resources in this example. The length of the array is equal to 11.

To create a random whale, two parts of the array are filled individually. The first part is filled firstly. If *j* is the random number generated for *ith* cell, then 1≤j≤k and i≠j. Then next n cells are filled. For each generated random number like *j*, we have 1≤j≤k. In fact, each resource is assigned to one gateway. The fitness function for each whale is equal to objective function (Equation (1)) which is explained in Section 3.1. The value of the fitness function is supposed to be decreased during the algorithm. In other word, whales with lower fitness value are better than other whales.

### 5.3. Distance Function

One of the most important functions used in the proposed algorithm, is the distance function which calculates the distance between two whales. Since the basic whale algorithm is a discrete algorithm and RA problem is a continuous problem, the concept of distance must be re-defined. All operators of the whale algorithm work based on the distance. So, the precise design of the distance function will have a great effect on the performance of the proposed algorithm.

Each whale is equivalent to a RA graph that has maximum k+n edges. k is the number of gateways and n is the number of resources. The distance between two whales is defined as the number of non-common edges in two RA graphs. Accordingly, the minimum distance is equal to zero and the maximum distance is equal to k+n. The pseudocode of the distance function is given in Algorithm 1.
**Algorithm 1:**$distance\;W_{1}\;,W_{2}$$\begin{array}{l} c=0;\\ for\;(i=1;\;i<k+n;\;i++)\\ \;\;\;\;\;\;\;\;\;\;\;\;\;\;\;\;\;\;\;if\;(W_{1}(i)\;\neq \;W_{2}(i))\\ \;\;\;\;\;\;\;\;\;\;\;\;\;\;\;\;\;\;\;\;\;\;\;\;\;\;\;\;\;\;\;\;\;\;\;\;\;\;c=c+1;\\ reture\;c; \end{array}$

Below, we show an example of how distance function works. There are 2 whales and 4 gateways in Table 3. The distance between these two whales is calculated based on the corresponding cells of the array that do not have the same value. These cells are shown in red. Accordingly, the distance between two whales is 5.

### 5.4. Spiral Function

To determine the type of each whale motion, a randomly generated number p is in [0, 1]. If this number is greater than half, whale motion is performed using spiral function which means spiral around the best whale. The concept of spiral must be re-defined for the RA problem. The pseudocode of the proposed spiral is given in Algorithm 2.
**Algorithm 2:**$spiral\;W_{i}\;round\;W_{best}$$\begin{array}{l} D=distance(W_{i},\;W_{best});\\ l=random\;value\;in\;[-1,\;1];\\ change=\left\lfloor \;D\;\times \;\cos\;(\;2\pi l)\right\rfloor ;\\ for(j=1;\;j<\left|change\right|;\;j++)\\ \;\;\;\;\;\;\;\;\;\;\;\;\;\;\;if(change>0)\\ \;\;\;\;\;\;\;\;\;\;\;\;\;\;\;\;\;\;\;\;\;\;\;\;\;set\;a\;random\;number\;in\;\;W_{i}(k)\;where\;W_{i}(k)\;=\;W_{best}\;(k);\\ \;\;\;\;\;\;\;\;\;\;\;\;\;\;\;elseif\;(change\;<\;0)\\ \;\;\;\;\;\;\;\;\;\;\;\;\;\;\;\;\;\;\;\;\;\;\;\;\;set\;a\;random\;number\;in\;W_{i}\;(k)\;\;where\;W_{i}\;(k)\;\neq \;W_{best}\;(k);\\ return\;W_{i} \end{array}$

In the above pseudocode, $W_{i}$ is a whale that should spiral and $W_{best}$ is the best whale. |change| denotes the number of entries of whale array that should be changed. If the value of change is positive, then entries having non-equal values are changed using $W_{best}$. Otherwise, entries having equal values are changed. Table 4 shows an example of spiral function for l=0.125 and D=5 in which change=⌊5×0.7⌋=3. Three changed entries are in red. Green entries show the distance.

### 5.5. Shrinking Function

At the beginning of each run of the algorithm, a random number p is generated. If this number if less than 0.5, another random number A should be considered. If A is less than 1, the shrinking function is performed. This function moves the current whale toward the best whale or prey. The difference between this function and spiral function is that, spiral function spirals around the prey while shrinking function moves directly and faster toward the prey. Hence, increasing the probability of executing the searchprey function at the end of algorithm is desirable.

The shrinking function adjusts its movement toward the prey based on the distance from the best whale. After calculating the distance, a percentage of non-equal entries is changed and moves toward the best whale with the probability of 50%. The pseudo-code of the shrinking function is shown in Algorithm 3.
**Algorithm 3:**$\;shrinking\;W_{i}\;to\;W_{best}$$\begin{array}{l} D=\;distance\;(W_{i},\;W_{best});\\ change\;=\left\lfloor A\;\times \;D\right\rfloor ;\\ for\;(j=0;\;j<\left|change\right|;\;j++)\\ \;\;\;\;\;\;\;\;\;\;\;\;\;k=a\;random\;index\;in\;W_{i}\;where\;W_{i}\;(k)\;\neq \;W_{best}\;(k);\\ \;\;\;\;\;\;\;\;\;\;\;\;\;rand\;=\;a\;random\;number\;in\;\left[0,\;1\right];\\ \;\;\;\;\;\;\;\;\;\;\;\;\;if\;(rand<\;0.5)\\ \;\;\;\;\;\;\;\;\;\;\;\;\;\;\;\;\;\;\;\;\;\;\;\;\;\;W_{i}\;(k)\;=\;W_{i}\;(best);\\ \;\;\;\;\;\;\;\;\;\;\;\;\;else\\ \;\;\;\;\;\;\;\;\;\;\;\;\;\;\;\;\;\;\;\;\;\;\;\;\;\;set\;a\;random\;number\;in\;W_{i}\;(k);\\ return\;W_{i} \end{array}$

Below, we show the shrinking function in an example. In Table 5, the distance between $W_{1}$ and $W_{best}$ is 4 which is shown in green. If A=0.5, then two entries from entries having non-equal values are changed. One of them become the best whale and the other one had a random change. These two changed entries are shown in red.

### 5.6. Searchprey Function

An important aspect of evolutionary algorithms is the ability to produce new solutions for the problem which prevents getting stuck in a local optimum. In the proposed method, the searchprey function is used for this purpose. The higher probability of performing this function at the beginning is desirable. Thus, the exploitation and optimization phase will start with a greater variety of solutions.

Searchprey function moves a whale toward a randomly selected whale. The basis of the movement is the distance between the two whales, but different methods have been used for motion as described in the following sections. The pseudocode of the searchprey function is given in Algorithm 4.

Four functions, shrinking, join, swap, and randomwalk, are used within this function. The shrinking function is explained in the previous section, with this difference that $W_{best}$ is used instead of $W_{j}$. The other three functions are described below.
**Algorithm 4:**$searchprey\;W_{i}$$\begin{array}{l} select\;W_{j}\;randomly\;from\;whale\;group;\\ D\;=\;distance\;(W_{i},\;W_{j});\\ change\;=\;\left|A\;\times \;D\right|;\\ rand\;=\;a\;random\;number\;in\;\left[0,\;1\right];\\ if\;(rand\;<\;0.25)\\ \;\;\;\;\;\;\;\;\;\;\;\;\;\;\;shrinking\;(W_{i},\;W_{j});\\ elseif\;(rand\;<\;0.5)\\ \;\;\;\;\;\;\;\;\;\;\;\;\;\;\;join\;(W_{i},\;W_{j},\;change);\\ elseif\;(rand\;<\;0.75)\\ \;\;\;\;\;\;\;\;\;\;\;\;\;\;\;swap\;(W_{i},\;W_{j});\\ else\\ \;\;\;\;\;\;\;\;\;\;\;\;\;\;\;randomwalk\;(W_{i},\;change);\\ return\;W_{i}; \end{array}$

#### 5.6.1. Join Function

The join function changes some parts of $W_{i}$ into the entries of $W_{j}$. The number of entries being changed is equal to |change|. The changing process can be sequential or random. After joining, whale $W_{i}$ get in in new position, part of which is inherited from $W_{j}$.

Table 6 shows an example of performing join function. In this example, $W_{1}$ moves toward $W_{2}$. The distance is 4 shown in green. Changed entries are selected linearly at the end of the array. These entries are shown in red.

#### 5.6.2. Swap Function

The swap function is the same as join function with this difference that the number of changed entries in join function is equal to |change|, while in swap function is exactly equal to the number of gateways ($\left|V_{g}\right|$) or the number of resources ($\left|V_{r}\right|$). In fact, in swap function, the new position of $W_{i}$ includes connection sub-graph of gateways in $W_{i}$ and connection sub-graph of resources in $W_{j}$ (and vice versa). The value of |change| has no effect on this function.

Table 7 shows an example of the swap function, in which $W_{1}$ moves toward $W_{2}$. The distance is 4 and shown in green and also has no effect on the result. Changed entries belong to the first part of the array which show the type of connection between gateways. These entries are shown in red.

#### 5.6.3. Randomwalk Function

Randomwalk function changes a number of entries of $W_{i}$ randomly. The number of entries being changed is equal to |change|. The values of whale $W_{j}$ have no effect on the generated numbers for $W_{i}$. The only effect of $W_{j}$ in running this function is the value of |change| that is generated based on the distance between $W_{i}$ and $W_{j}$.

In fact, randomwalk function produces a completely random movement in $W_{i}$ and takes the whale to a new position. So, it is likely to discover new solutions after performing this function.

### 5.7. Parameter a

One of the most important parameters in the proposed method is a. The value of A which determines the type of executing function in any steps of the algorithm is calculated based on parameter a according to Equation (14).

(14) A=2ar−a

The value of a is considered between 2 to 0 in descending form. So, at the beginning of the algorithm it is likely that the value of A is greater than one and the searchprey function is executed. As the value of a decreases, the value of A also becomes less than one in most cases. Therefor shrinking function will be executed. In this way, after the discovery of new solutions, the algorithm enters the exploitation phase. In fact, decreasing the value of a helps to narrow the spiral movement around the prey and whales focus on optimizing the final solution.

### 5.8. Graph Clustering

The quality of the initial whales has a great impact on the performance of the whale algorithm. For this reason, in the proposed method a heuristic clustering algorithm has been used in order to increase the quality of the initial solutions. With the help of this method, solutions with high optimization capability would be produced and more likely give us better solutions. The pseudo code of the proposed clustering method is shown in Algorithm 5. In this method, gateways are scanned in sequence, and in each round, the nearest resource is assigned to that gateway. This will continue until the end of resources.
**Algorithm 5:**GraphClustering$\begin{array}{l} Input:\;Gateway\;and\;Resource\;Communication\;Cost\;Matrix\\ Output:\;A\;solution\;for \end{array}$$\begin{array}{l} selectedResources\;=\;\{\};\\ whale=zero\;array\;in\;length\;\left|G\right|\;+\;\left|R\right|;\\ while\;(selectedResources\;\neq \;Resources)\;\;\{\\ \;\;\;\;\;\;\;\;\;\;\;\;\;foreach\;Gateway\;like\;G_{i}\;\;\{\\ \;\;\;\;\;\;\;\;\;\;\;\;\;\;\;\;\;\;\;\;\;\;\;\;\;find\;R_{j}\;as\;a\;Resource\;with\;lowest\;cost\;for\;G_{i};\\ \;\;\;\;\;\;\;\;\;\;\;\;\;\;\;\;\;\;\;\;\;\;\;\;\;assign\;R_{i}\;to\;G_{i}\;in\;whale;\\ \;\;\;\;\;\;\;\;\;\;\;\;\;\;\}\\ \}\\ set\;Gateway\;randomly\;in\;whale;\\ return\;whale; \end{array}$

**Figure 3:**
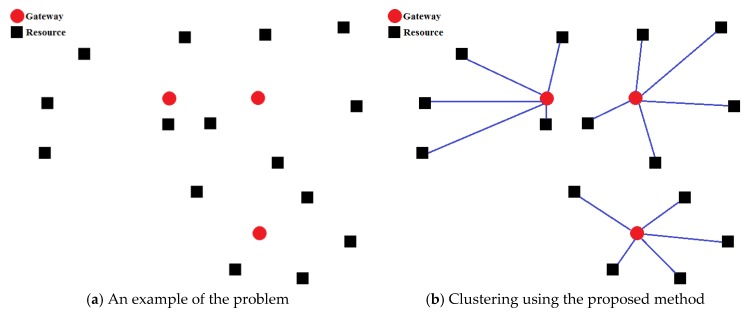
Graph clustering.

In the following, we describe how this method works by an example. In the example of the Figure 3a, red circles represent gateways and black squares represent resources. There are three gateways and fifteen resources. The distance between nodes is plotted based on their communication cost. As shown in Figure 3b, fifteen resources are divided between Gateways accordingly. In each round, the nearest resource is considered for each node. The output of this algorithm varies according to the order in which the gateways are selected, so by scanning gateways in different orders, various solutions can be produced for the problem. The point of this graph clustering method is that the penalty of the produced solutions is equal to zero, because it fully observes load balancing.

## 6. Implementation and Analysis

This section provides the simulation of the proposed algorithm and then evaluates its performance. Simulation is done in MATLAB software environment on a desktop computer. Given that there is no processing time in the evaluations, it is not necessary to introduce the computer specifications and software version.

None of the prior researches on the RA problem in cloud computing used a common and public dataset. Creating a dataset covers all conditions of the scheduling problem. Given that data are not the same, we re-implemented some of them in order to compare with previous works. Apart from these datasets, RA is an administrative problem which have to be solved before deployment of the network. Since solving the problem is not a real-time activity, there is enough room to process and reach the BS. Hence, the network admin could easily execute the proposed method with the network data and decide on the assignment pattern.

### 6.1. Dataset

The test data are generated in small, medium and large size. The number of gateways varies from 4 to 100 and the number of resources varies from 10 to 800. A total of eight test samples are designed for the proposed method. The list of sample tests is shown in Table 8.

In Table 8, three datasets $DS_{1}$, $DS_{2}$, and $DS_{3}$ are small size, two datasets $DS_{4}$ and $DS_{5}$ are medium size, and datasets $DS_{7}$ and $DS_{8}$ are large size. Regardless of scale, the cost of communication between resources and gateways is a random number ∈[1.20] and the cost of communication between gateways is a random number ∈[20.40]. The cost of connection between gateways is always higher than the cost of connection between resources and gateways.

**Figure 4:**
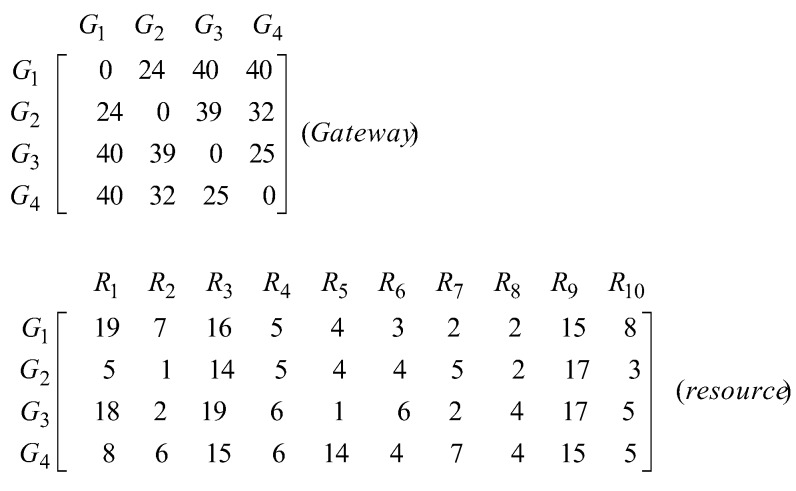
Dataset $DS_{1}$.

Each dataset has two matrices. If k is defined as the number of gateways and n denotes the number of resources, then one of these matrices is a symmetric matrix called $Gateway_{k\times k}$ of size k×k which shows the cost of communication between gateways and the other one is a matrix called $Resource_{k\times n}$ of size k×n which shows the cost of connection between resources and gateways. You can see the dataset $DS_{1}$ in Figure 4.

### 6.2. Simulation Results

We applied the proposed algorithm on the generated dataset and in order to compare with other methods, we also applied two methods GA [36] and Search Economics for IoT Resource Allocation (SEIRA) [16] on this data. The main criterion is the comparison of different methods of RA problem is $T_{c}$ (the total cost of the messages in RA).

Table 9 shows the results of the proposed algorithm in comparison with previous methods. Given the fact that data is randomly generated and the RA problem is a Np-complete problem, the optimal solution is not available and only the results of different methods can be used to compare them. For the results shown in Table 9, the proposed algorithm has been executed for 10,000 and 20,000 times. Other methods are also executed for the relatively same initial population and rounds in order to have fair results.

The results of the simulation of the proposed algorithm and its comparison with the other two algorithms indicate that whale algorithm is good at solving RA problem. Obtained results for whale algorithm, most of the time are better than GA. In comparison with the SEIRA algorithm, the whale algorithm has the same results and in some cases better results.

**Figure 5:**
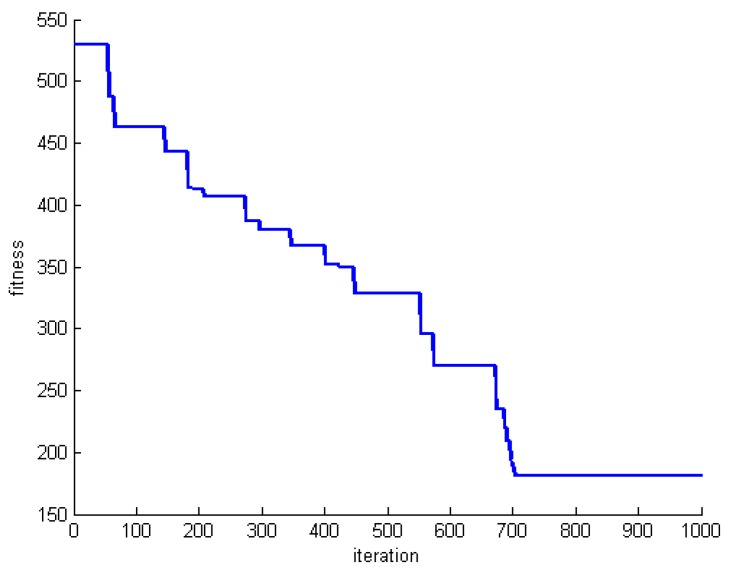
The fitness of the best whale during whales’ motion in $DS_{1}$.

**Figure 6:**
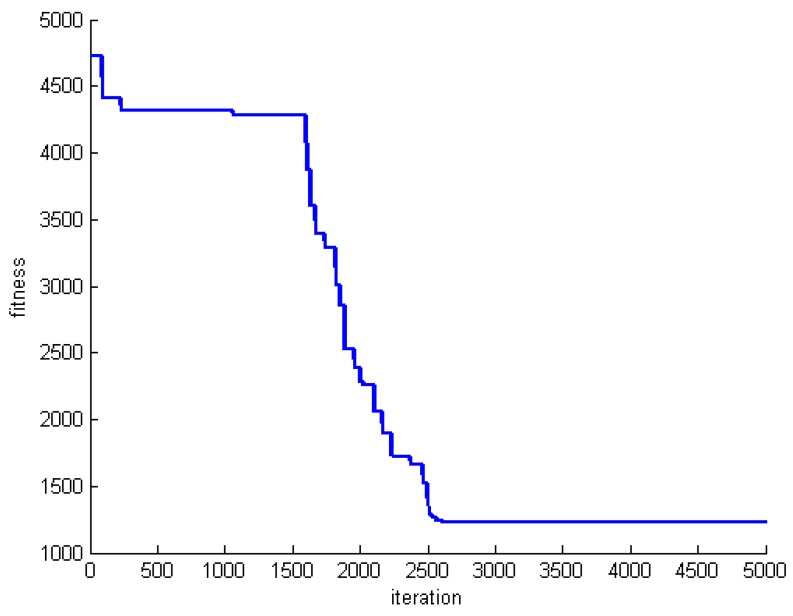
The fitness of the best whale during whales’ motion in $DS_{5}$.

**Figure 7:**
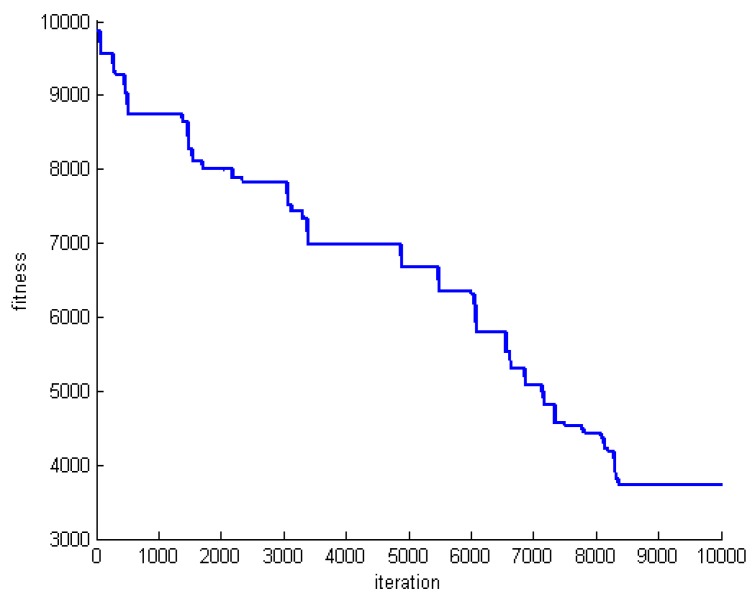
The fitness of the best whale during whales’ motion in $DS_{8}$.

One criterion to evaluate the performance of the proposed algorithm is convergence speed. To do this, we save the fitness of the best whale in each round of whale motions. Figure 5, Figure 6 and Figure 7 show the fitness of three datasets $DS_{1}$, $DS_{5}$, and $DS_{8}$.

**Figure 8:**
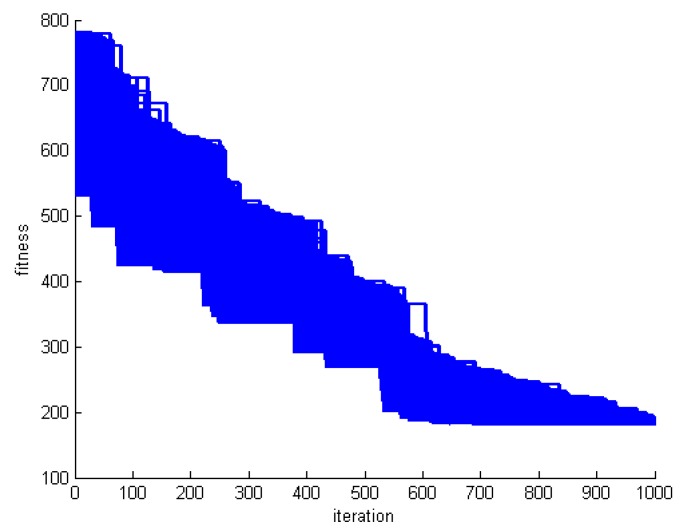
Whale fitness during motion in $DS_{1}$.

**Figure 9:**
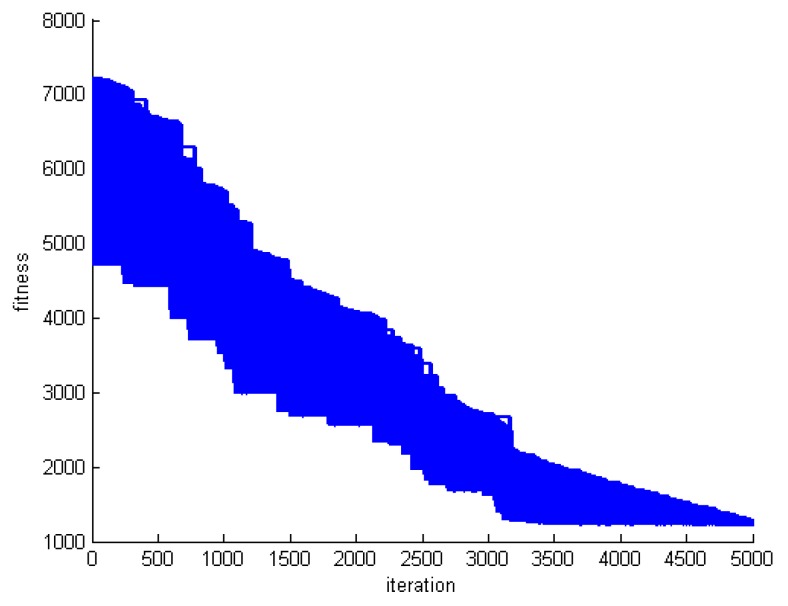
Whale fitness during motion in $DS_{5}$.

**Figure 10:**
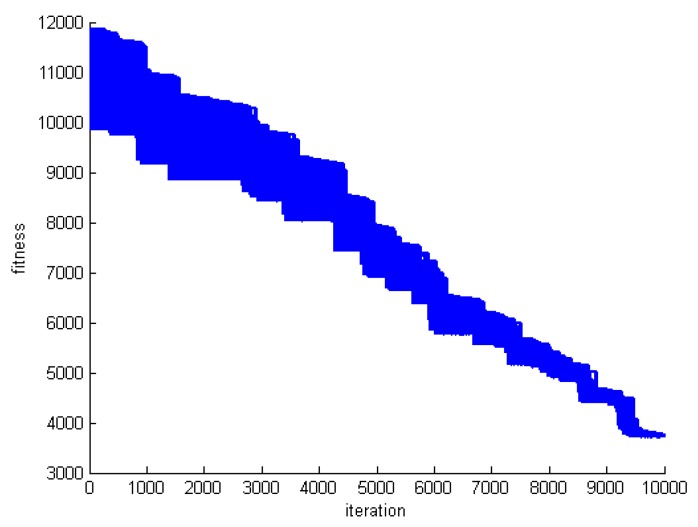
Whale fitness during motion in $DS_{8}$.

The whale algorithm is inspired by the group haunting of whales. Hence, in each round of the proposed algorithm, all whales should approach the prey. From the RA problem solving point of view, it means that, all whales change over time. Figure 8, Figure 9 and Figure 10 show the fitness of all whales for three datasets $DS_{1}$, $DS_{5}$, and $DS_{8}$.

### 6.3. The Effect of Spiral, Shrinking and Searchprey Functions

One of the most important points to be considered in the proposed method is the efficiency of the designed functions. Three main functions named spiral, shrinking and searchprey functions have been designed in the proposed method. The spiral function is designed to spiral around the BS (or prey). The shrinking function moves a whale directly toward the best whale and searchprey function moves a whale toward a random whale. The first and second functions are used to optimize the existing solutions and the third one is used to discover new solutions.

If the designed functions do not have the necessary performance, then the proposed algorithm has the same performance as a random algorithm which produces many random solutions for the problem. In contrast, the high efficiency of these functions makes it possible to quickly converge to an optimum solution. To investigate this issue, we ran the proposed algorithm with different numbers of whales and motions. In all runs, the multiplication of the number of whales in the number of steps is a constant number. That is, in all simulations, the same number of solutions is produced for the problem. The results of three datasets $DS_{1}$, $DS_{5}$ and $DS_{8}$ are shown in Table 10, Table 11 and Table 12 respectively.

The results of the three above tables indicate that, using three main functions to produce solution yields to better solution than random ones. In contrast, if we increase the number of rounds and reduce the number of whales, we will not get good results. We must balance between the number of whales and the number of rounds.

### 6.4. The Effect of Factor a

One of the most important factors in whale algorithm is a. The intelligent change of the amount of this factor during the execution of the algorithm has a great influence on the performance of the algorithm. The whale algorithm has two phases. The first one is the discovery phase in which the algorithm should act in such a way that new solutions rare generated randomly. In fact, in this phase produced points are supposed to be distributed uniformly in the problem space. The first phase begins with the start of the algorithm and then algorithm enters the second phase. The second phase is the exploitation phase in which the algorithm acts to optimize the previously generated solutions. In other words, in this phase, instead of producing new random points, we are looking for optimal points around the points that were previously generated. The second phase starts from the middle of the algorithm and continues until the end.

Factor a determines that if the algorithm is in the discovery phase or exploitation phase. So the intelligent change of this factor during the execution of the algorithm changes the discovery phase logically and slowly into the exploitation phase. The value of the a varies in descending order in [2, 0] according to Equation (15). In this equation, *G* is the number of whale’s motion and *i* is the current execution of the algorithm.

(15) $$a=2\times {\left(\frac{G-i+1}{G}\right)}^{2}\;$$

**Figure 11:**
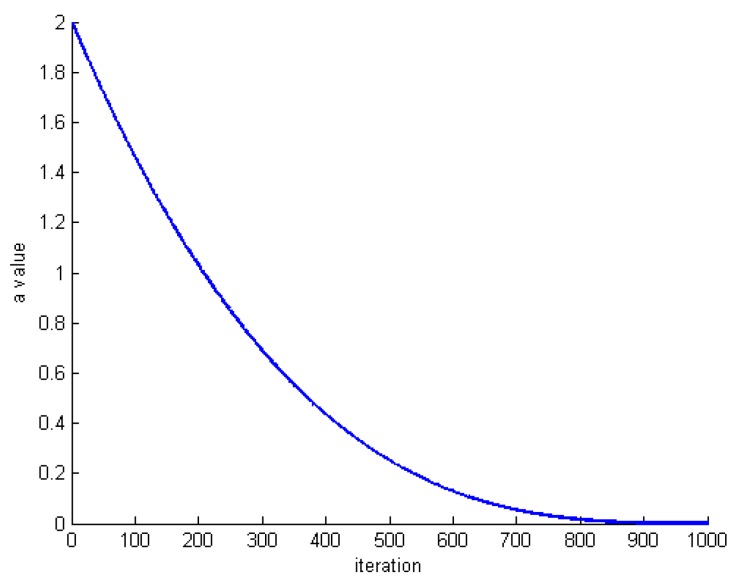
Changes of the factor a for G=1000.

Figure 11 shows the changes of factor a for G=1000.

To better examine the effect of a on the execution of the algorithm, we examine the proposed method in two modes. First, a state in which a hold the algorithm in discovery phase with a value close to 0 and then a state in which the algorithm begins with exploitation phase with a value close to 0 for a.

**Figure 12:**
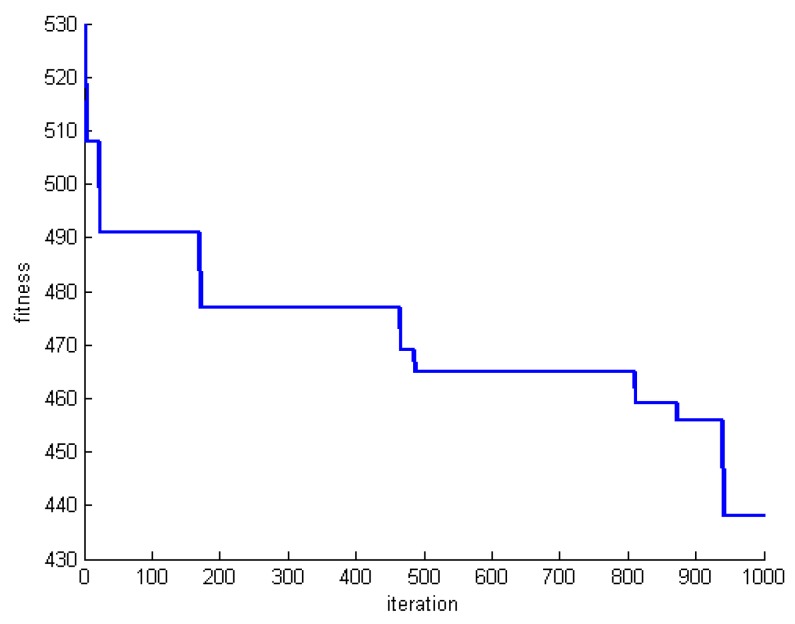
Changes of the best whale for a=2 in $DS_{1}$.

**Figure 13:**
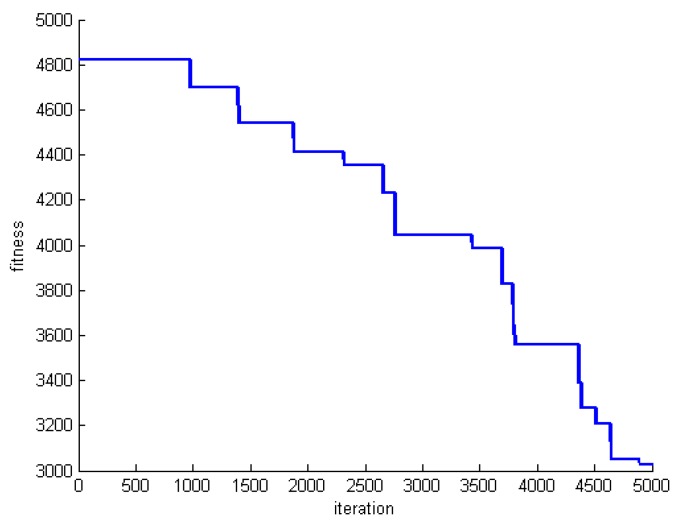
Changes of the best whale for a=2 in $DS_{5}$.

**Figure 14:**
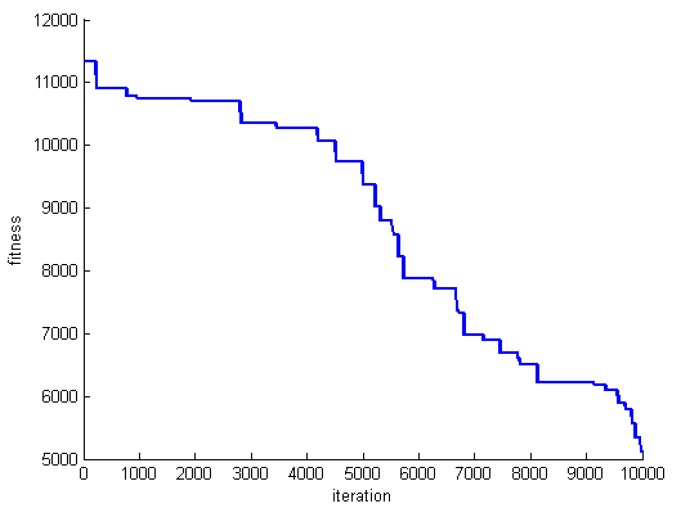
Changes of the best whale for a=2 in $DS_{8}$.

Figure 12, Figure 13 and Figure 14 show the changes of the best whale for a=2. Both the number of whales and rounds are set to 1000. Experiments are performed for datasets $DS_{1}$, $DS_{5}$, and $DS_{8}$. Focusing on the results reveal that the obtained results are far from the optimum solutions. In addition, the process of optimization is very slow and random.

**Figure 15:**
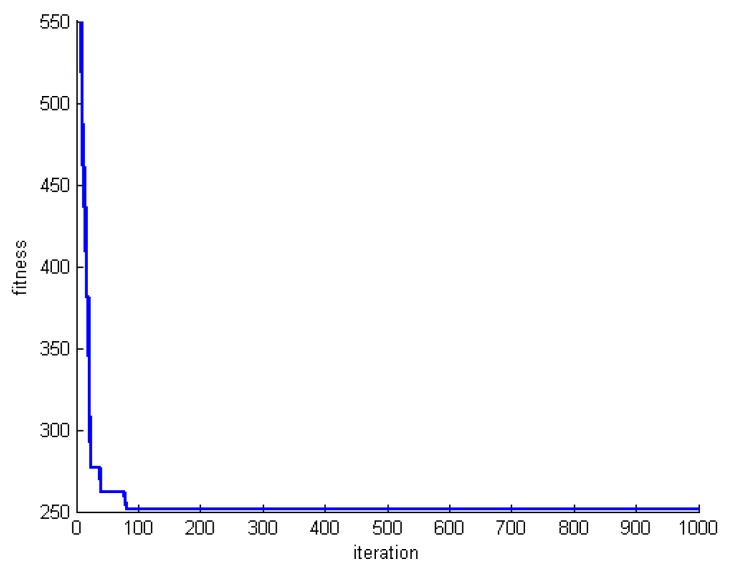
Changes of the best whale for a=0 in $DS_{1}$.

**Figure 16:**
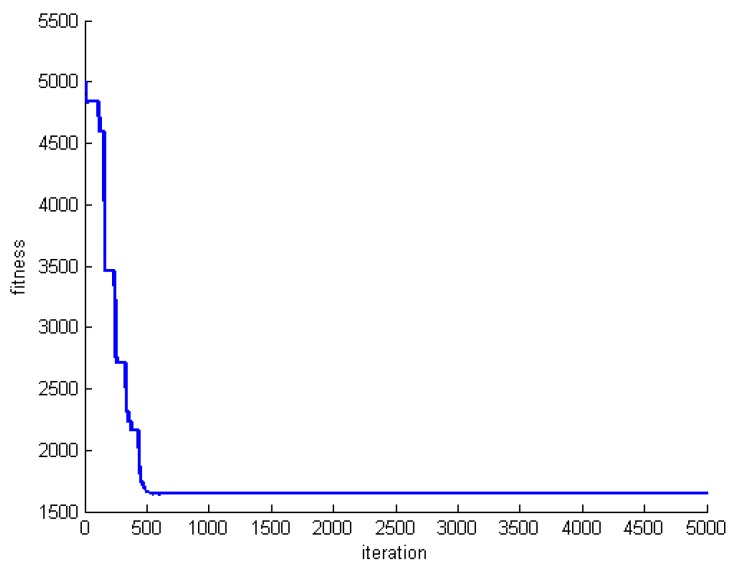
Changes of the best whale for a=0 in $DS_{5}$.

**Figure 17:**
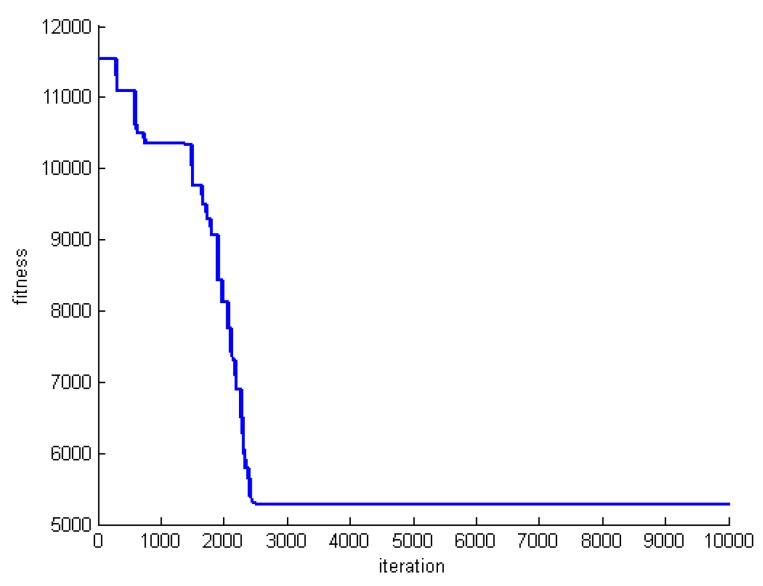
Changes of the best whale for a=0 in $DS_{8}$.

Figure 15, Figure 16 and Figure 17 show the changes of the best whale for a=0. Both the number of whales and rounds are set to 1000. Experiments are performed for datasets $DS_{1}$, $DS_{5}$, and $DS_{8}$.

Focusing on the results reveal that initial whales are optimized quickly, and the algorithm does not produce a better solution anymore. In fact, the algorithm is stuck in the local optimum.

### 6.5. The Effect of the Graph Clustering

One of the methods used to reinforce the proposed algorithm is graph clustering. This method produces initial solutions with better quality and thus enhances the optimization process. To investigate the effect of the clustering in the proposed method, we must run the algorithm without applying the graph clustering method and using fully random initial solutions, and then compare the results with that of the complete algorithm. The result of this comparison is shown in Table 13. The results of this table show that, whatever the size of the problem is larger, the effect of the graph clustering is greater.

**Figure 18:**
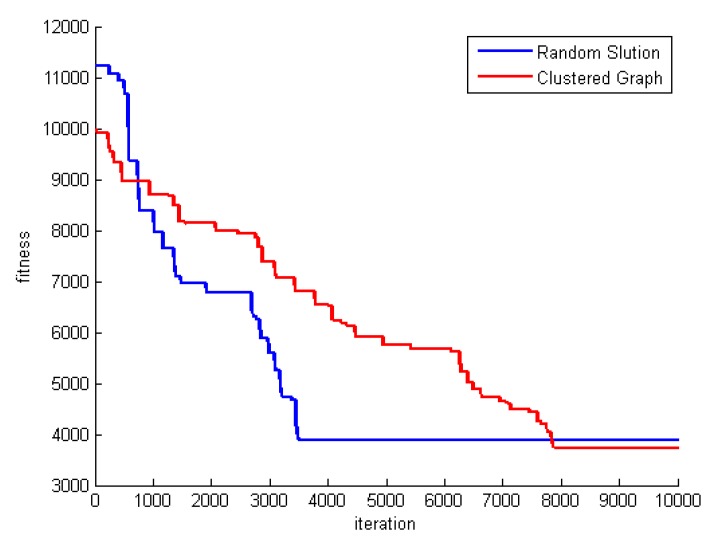
The effect of graph clustering on convergence.

One of the other effects of the graph clustering is the convergence rate. To investigate the effect of graph clustering on the convergence rate, the process of the best fitness for problem DS8 is shown in Figure 18. As seen in this figure, in the case of using clustering, convergence rate is lower. The reason is getting stuck in local optimum in the random solution mode. However, ultimately, the method that uses graph clustering produces better solutions. The reason for producing a better initial generation using clustering is that it has high optimization capability.

## 7. Conclusions

Cloud computing is a popular model for users of cloud resources because of the “pay-per-use” model. However, the RA problem is one of the most important challenges in this kind of system due to the high volume of resources and user requests, which has attracted many researchers to the field. RA and scheduling refers to a method for allocating resources to users and the main objective of a schedule is reducing runtime and allocating optimal resources to tasks. On one hand, the improper use of resources leads to increased energy consumption, and as a result, environmental warming. Therefore, task scheduling and RA in large systems such as cloud computing are also important besides runtime and can’t be ignored. In this paper, a novel algorithm based on whale optimization is proposed with the aim of reducing runtime of tasks and allocating optimal resources to tasks. The proposed method suggested a discrete definition for the whale algorithm in order to optimize RA problem in cloud environment. Each whale is designed in the form of an array. Based on this array, a new concept of distance is defined for the distance function. This function is very useful for the whale algorithm. The spiral function is designed for spiral movement and shrinking and searchprey functions are designed for direct movement. The results obtained show that the proposed whale algorithm has a high ability to solve the RA problem in the cloud. The functions designed for base operators of the whale algorithm have been able to search the solution space of the problem well and found reasonable solutions for the mentioned problem by optimizing the discovered solutions. An important point in the correct execution of the algorithm is the logical adjustment of the value of the a which enters the algorithm from the discovery phase to the exploitation phase.

## Figures and Tables

**Table 1:** A summary of related works.

Reference	Algorithm	Year	Pros and Cons
[31]	Consensus-based approach	2014	- Easy implementation - Not useful for big problems
[34]	Asymptotic shapely value-based resource allocation scheme	2016	- Deterministic approach - high complexity
[36]	Genetic Algorithm	2015	- Efficient for big problems - Unable to find optimal solution
[16]	Search Economics Algorithm and k-means clustering algorithm	2018	- Producing better solutions in first generation - Time consuming algorithm
[47]	Fuzzy based job classification	2017	- Considering priority for resources - Not suitable for every environment

**Table 2:** A whale with 4 gateways and 7 resources.

	Gateway				Resource						
Indices	1	2	3	4	5 ($r_{1}$)	6 ($r_{2}$)	7 ($r_{3}$)	8 ($r_{4}$)	9 ($r_{5}$)	10 ($r_{6}$)	11 ($r_{7}$)
Allocate	3	1	4	3	1	4	4	4	3	2	1

**Table 3:** The distance between two whales.

	Gateway				Resource						
Indices	1	2	3	4	5 ($r_{1}$)	6 ($r_{2}$)	7 ($r_{3}$)	8 ($r_{4}$)	9 ($r_{5}$)	10 ($r_{6}$)	11 ($r_{7}$)
W1	3	1	4	3	1	4	4	4	3	2	1
W2	3	3	4	2	1	2	2	4	3	4	1

**Table 4:** An example of spiral function.

	Gateway				Resource						
Indices	1	2	3	4	5 ($r_{1}$)	6 ($r_{2}$)	7 ($r_{3}$)	8 ($r_{4}$)	9 ($r_{5}$)	10 ($r_{6}$)	11 ($r_{7}$)
W1	3	1	4	3	1	4	4	4	3	2	1
Best	3	3	4	2	1	2	2	4	3	4	1
Spiral	3	1	4	1	1	2	4	4	3	4	1

**Table 5:** An example shrinking function.

	Gateway				Resource						
Indices	1	2	3	4	5 ($r_{1}$)	6 ($r_{2}$)	7 ($r_{3}$)	8 ($r_{4}$)	9 ($r_{5}$)	10 ($r_{6}$)	11 ($r_{7}$)
W1	3	1	4	3	1	4	4	4	3	2	1
Best	3	1	4	2	1	2	2	4	3	4	1
Shrinking	3	1	4	2	1	3	4	4	3	2	1

**Table 6:** An example of join function.

	Gateway				Resource						
Indices	1	2	3	4	5 ($r_{1}$)	6 ($r_{2}$)	7 ($r_{3}$)	8 ($r_{4}$)	9 ($r_{5}$)	10 ($r_{6}$)	11 ($r_{7}$)
W1	3	1	4	3	1	4	4	4	3	2	1
W2	3	1	4	2	1	2	4	4	2	4	1
Join	3	1	4	3	1	3	4	4	2	4	1

**Table 7:** An example of swap function.

	Gateway				Resource						
Indices	1	2	3	4	5 ($r_{1}$)	6 ($r_{2}$)	7 ($r_{3}$)	8 ($r_{4}$)	9 ($r_{5}$)	10 ($r_{6}$)	11 ($r_{7}$)
W1	3	1	4	3	1	4	4	4	3	2	1
W2	3	3	4	2	1	4	4	4	2	4	1
Swap	3	3	4	2	1	4	4	4	3	2	1

**Table 8:** Data set list.

Data Set Name	Number of Gateways	Number of Resources
DS1	4	10
DS2	4	12
DS3	4	16
DS4	40	100
DS5	40	200
DS6	40	400
DS7	100	400
DS8	100	800

**Table 9:** Result and comparison.

	GA [36]	SEIRA [16]	WOA (Proposed)
DS1	180.5	180.5	180.5
DS2	214	214	214
DS3	242.5	242.5	242.5
DS4	841	835	836.6
DS5	1260.4	1233	1230
DS6	1920.6	1905.3	1901.4
DS7	2728.8	2669.1	2661.8
DS8	3857.3	3753.7	3725

**Table 10:** The best solution of $DS_{1}$ for different modes.

Number of Whales	Number of Rounds	Best Solution
1,000,000	1	270.5
100,000	10	233
10,000	100	197.5
1000	1000	180.5

**Table 11:** The best solution of $DS_{5}$ for different modes.

Number of Whales	Number of Rounds	Best Solution
25,000,000	1	1470.1
500,000	50	1431.4
50,000	500	1298.8
5000	5000	1230

**Table 12:** The best solution of $DS_{8}$ for different modes.

Number of Whales	Number of Rounds	Best Solution
100,000,000	1	4357
10,000,000	10	4124.5
1,000,000	100	4023.2
10,000	10,000	3725

**Table 13:** Comparison of running algorithm in clustering and random mode.

Problem	Graph Clustering		Random	
	Best Answer in First Generation	The Final Answer	Best Answer in First Generation	The Final Answer
DS1	540	180.5	571	180.5
DS2	612	214	664.5	214
DS3	707.2	242.5	755	242.5
DS4	2927	836.6	3374	852
DS5	4750	1230	5219.4	1258.5
DS6	6206	1901.4	7126	1942
DS7	7480.2	2661.8	9135	2730.2
DS8	9955	3725	11,324	3892
